# The World’s Columbian Dental Congress

**Published:** 1893-10

**Authors:** 


### Editorial.

### THE WORLD'S COLUMBIAN DENTAL CONGRESS.

It is always difficult to arrive at satisfactory conclusions regarding the result of the deliberations of a large body assembled in any given capacity, and doubly so when this convocation of persons has met together ostensibly for a special scientific purpose. The great variety of mind to reach and satisfy not only renders it necessary to have the proceedings cover a wide scope, but it also naturally causes a diversity of sentiment regarding the ultimate value attained.

That this was true of the recent Congress must be conceded by all those present at its deliberations. It was to all intents and purposes a mass-meeting of the dental profession, and as such can lay no claim to have been a scientific body. It was organized for a special work, to bring dentists from all parts of the world in unison for a limited period, and if it served a purpose in measurably breaking down the bars of prejudice occasioned by nationalities, various languages, isolated conditions, and crude conceptions of professional work, it, perhaps, accomplished as much as could reasonably be expected.

That this view was not the one hoped for must be acknowledged. It was anticipated that the sessions of this Congress would mark an epoch in scientific dentistry, and probably lead to more extended gatherings of a similar character on great occasions in other parts of the world. Whether this will be the result time alone can determine.

The hour has not yet arrived to review the work of this Congress or to do justice to it, for the official record is not before us, and while that made by the faithful corps of reporters is satisfactory, it does not embody all the work laid out and probably completed by the committees.

While this is recognized, it does not absolve the conductors of journals representing the profession from a duty pressing upon them to give their views regarding the work performed and the possible results.

We felt called to repeatedly urge upon the committees in charge the importance of making this Congress truly a scientific convention, and we have also at times freely criticised what, in our judgment, seemed to endanger the success of the undertaking. We felt, in common with the best thought of the dental profession, that this Congress should be an epoch-making gathering, and in order to accomplish this it should, in every sense, represent the best scientific mind of the world. That this view was not accepted by the committee in charge was, in our opinion, the first fatal step which led, by a series of mistakes, down to a conclusion not creditable to American dentistry.

It is not desirable at this late day to recur to the lamentable error of the earlier organization. That first false step led to subsequent mistakes, until the final organization of the Congress, which, to our view, was the most open to criticism of any that had preceded it. It early became apparent that this Congress could not be anything more than an aggregation of uncertain elements. No rules were elaborated in advance to prevent the entrance of undesirable persons, and the means subsequently adopted to attain this end were not calculated to effect the result. There was some effort made to discover the professional standing of certain parties, but in the rush of applications for membership this practically amounted to nothing, and seemed to be finally abandoned, as all who applied and could pay the amount demanded were furnished with a certificate.

The committee on the organization of this Congress was appointed by the American Dental and Southern Dental Associations to arrange the preliminary work of this meeting. The original resolution was adopted at Excelsior Springs, Missouri, in 1890, and the following is the essential part, italics ours: "When this committee is so completed it shall be clothed with full power to take such action as it in its judgment may deem best for *creating an organization* for the purpose of holding a dental meeting in Chicago in 1893." It was never contemplated by the former body, for we cannot speak for the latter, that this work should extend beyond the preliminary organization, and that accomplished, the labors of the committee practically ended. This, however, was evidently not the idea entertained by this body. It was assumed that it possessed not only full power to organize, but was to be in all essentials *the* Congress. It made the rules for the government of the latter, prescribed how these should be carried out, and refused absolutely to permit any one even to suggest a modification. In a word, the only privilege they accorded was to sit in the general meeting and take part in the discussions of the sections.

The arrangement of the Congress into one general meeting at twelve o'clock (noon), and into eight sections in the afternoon, was another almost fatal mistake. The explanation was that the work could not be accomplished in one general meeting. It was clearly apparent that all interest would be sacrificed by this subdivision, but as no one was permitted to find fault with the arrangement, it was acceded to as the only thing possible under the circumstances.

The excuse that there would not have been time to have read and discussed all the papers would have been a valid one if all such presented had been worthy of this Congress; but they were not.

The Committee on Essays had, doubtless, an unpleasant task before it, but, disagreeable as it was, the duty of selection should have been performed without fear or favor. It would seem from the result that it concluded to accept everything presented, good, bad, and indifferent, and let the Congress be the judge of their merits. The meetings were, therefore, served with a singular mixture of a few good papers, some of doubtful value, and a large number which would discredit any small local society. An analysis of fifty-seven papers presented to the Congress, exclusive of addresses, gives, in the judgment of the writer, the following result: Fourteen which can claim a place as worthy of consideration, among these several of rare scientific value, seventeen fairly good articles, and twenty-three that should have had no place at such a meeting. Three others were admitted after they had been repeatedly read, at least in substance, before societies and published in the journals. If the twenty-six papers had been rejected, and the convention called together at 10 A.M., ample time would have been given to have had them read and considered in a dignified manner. Washington Hall would have comfortably accommodated the entire body, and as there is always life in numbers, the discussions would have been varied in character and valuable in results. That such would have been the case was demonstrated in other congresses held in the same building, where this subdivision was not attempted.

While it is not possible to call a congress an absolute failure where over a thousand dentists assemble from all parts of the world, this one cannot be called a great success, and for the reasons given. Outside of the professed enthusiasm over the result manifested in certain journals, there is a universal feeling of disappointment.

The arrangement for clinics was beneath criticism, and we shall not attempt it. The operations, to the few, may have been valuable, but for the majority to see them was impossible, while the intolerable atmosphere, coupled with the noise made by the officials, rendered it simply unbearable. This portion of the programme could have been omitted without serious detriment to the Congress, and with gain to the reputation of those who managed this portion of the work.

Whatever of good this Congress accomplished will be made manifest in the published transactions, but we certainly feel assured that if international congresses are to be a part of the programme of the future the mistakes of this must be avoided. The only true course to prevent difficulty is to have international conventions delegated bodies, and when once convened let the control be in the hands of those sent as representatives. This kind of an international congress, conducted on scientific lines, must commend itself to all right-thinking minds, but it is hoped that international mass-meetings will end with the World's Columbian Dental Congress of 1893.

